# Clinical Features and Treatment Outcomes of Patients with Drug-Resistant and Drug-Sensitive Tuberculosis: A Historical Cohort Study in Porto Alegre, Brazil

**DOI:** 10.1371/journal.pone.0160109

**Published:** 2016-08-09

**Authors:** Vania Celina Dezoti Micheletti, Afrânio Lineu Kritski, José Ueleres Braga

**Affiliations:** 1 Postgraduate School of Pulmonology, Universidade Federal do Rio Grande do Sul, Porto Alegre, RS, Brazil; 2 School of Medicine, Universidade Federal do Rio de Janeiro, Rio de Janeiro, RJ, Brazil; 3 Department of Collective Health, Universidade Estadual do Rio de Janeiro; Public Health Researcher, Oswaldo Cruz Foundation, Rio de Janeiro, RJ, Brazil; University of Cape Town, SOUTH AFRICA

## Abstract

**Purpose:**

To evaluate the clinical features and treatment outcomes of patients with pulmonary tuberculosis, stratified by level of drug resistance.

**Methods:**

This was a historical cohort study based on data from the II National Anti-Tuberculosis Drug Resistance Survey (2006–2007) collected at eight participating health care facilities in Porto Alegre, southern Brazil. The cohort was followed for 3 years after the start of treatment.

**Results:**

Of 299 cases of smear-positive pulmonary tuberculosis included in the study, 216 (72.2%) were diagnosed at five public primary health care units and 83 (27.8%) at three public hospitals. Among these cases, the prevalence of drug-resistant tuberculosis was 14.4%, and that of multidrug-resistant tuberculosis was 4.7%. Overall, 32.0% of drug-resistant and 2.0% of multidrug-resistant cases occurred in previously treated patients. The most common comorbidity in the sample was HIV infection (26.2%). There was no association between drug-resistant or multidrug-resistant tuberculosis and sociodemographic variables. Cure was achieved in 66.7% of patients, and the default rate was 21.2%. The 2-month sputum conversion rate was 34.2%, and the relapse rate was 16.9%. Patients with drug-resistant tuberculosis had lower rates of cure (45.2%) and 2-month sputum conversion (25%), as well as a higher relapse rate (30.7%).

**Conclusion:**

These results highlight the urgent need for a more effective TB control program in this geographical setting, with a major emphasis on treatment of drug-resistant and multidrug-resistant tuberculosis.

## Introduction

Tuberculosis (TB) has been considered a worldwide public health problem by the World Health Organization (WHO) since 1993, and global actions have been taken to control this disease [1,2]. However, in recent years, epidemiological indicators have pointed to a low effectiveness of TB prevention and control activities in regions where HIV rates are high and where drug-resistant (DR), multidrug-resistant (MDR), or extensively drug-resistant (XDR) TB has been identified [3–5]. Cure rates of only 58% to 67% have been achieved in these settings, according to systematic reviews and meta-analyses [3–5].

Since the launch of the Global Project on Anti-Tuberculosis Drug Resistance Surveillance in 1994 by WHO, a large volume of drug resistance data from several countries has been collected and analyzed [3]. However, drug resistance surveys have provided little information on clinical and laboratory outcomes after initiation of treatment in patients with a diagnosis of DR-TB or MDR-TB [6].

In Brazil, the effectiveness of TB control programs tends to be lower in major cities. Preliminary data obtained from the II National Anti-Tuberculosis Drug Resistance Survey conducted between 2006 and 2007, involving 4,421 patients from seven states (Rio de Janeiro, Rio Grande do Sul, Bahia, Distrito Federal, Santa Catarina, Minas Gerais, and São Paulo), show rates of 1.4% (1.0–1.8) for primary MDR-TB and 7.5% (5.7–9.9) for acquired MDR-TB. In Porto Alegre, a large city and the capital of the Southern Brazilian state of Rio Grande do Sul, primary and acquired MDR-TB rates were higher than the national average at 2.2% and 12.0% respectively [7]. This rate for primary MDR-TB is also above the upper limit of 2% established by WHO [1]. In addition, high rates of TB and HIV co-infection (35.0%) and treatment default (19.6%) have also been reported in this geographical setting [8].

The purpose of this study was to evaluate the clinical features and treatment outcomes of DR-TB and MDR-TB cases in Porto Alegre, southern Brazil, as identified through the nationwide survey.

## Methods

This was a historical cohort study based on data from the II National Anti-Tuberculosis Drug Resistance Survey conducted in 2006 and 2007 in Brazil. We analyzed the data collected at the eight public health care facilities (five primary health care units and three hospitals) that participated in the survey in Porto Alegre. Porto Alegre is the capital of Rio Grande do Sul, the southernmost state of Brazil, and had a population of 1,415,237 inhabitants and a Human Development Index of 0.865 in 2007 [9]. This study was approved by the Porto Alegre Municipal Health Department Research Ethics Committee (protocol no. 001.053413.05.3). Written informed consent was obtained from all participants or a legally authorized representative prior to their inclusion in the study.

The II National Anti-Tuberculosis Drug Resistance Survey was performed by means of in-person standardized interviews conducted by trained interviewers, who used an instrument with pre-coded response categories [10].

Participant samples were analyzed by the Löwenstein–Jensen proportion method, in accordance with Brazilian National Tuberculosis Guidelines [8]. All clinical samples were sent to the Rio Grande do Sul State Referral Laboratory for culture, drug susceptibility testing, and identification at the species level. Tests were performed as per standard laboratory procedures; the techniques employed are described elsewhere [11, 12]. Smears were stained by the Ziehl–Neelsen method at the local Mycobacteriology Laboratory and scored as per international guidelines. All laboratories involved in testing used a double-blinded method for internal quality control. In addition, all samples identified as drug-resistant were retested by another referral laboratory, as were 15% of those identified as susceptible.

WHO definitions of drug sensitivity and drug resistance were used. Namely, DS-TB was defined as TB caused by strains of *Mycobacterium tuberculosis* that are sensitive to any anti-TB drug; DR-TB, as TB caused by strains that are resistant to at least one drug; monoresistant TB, as TB caused by strains that are resistant to one drug; and MDR-TB, as TB caused by strains of *M*. *tuberculosis* that are resistant to at least isoniazid and rifampicin [1].

A total of 299 TB patients with DR-TB or MDR-TB were identified at the participating public health care facilities in Porto Alegre. Of these, 216 (72.2%) cases were diagnosed at five public primary health care units and 83 (27.8%) at three public hospitals. Patient care during the study did not deviate from routine procedures.

The following variables collected during the nationwide survey were analyzed in the present study: sociodemographic characteristics, clinical features, comorbidities, and clinical and laboratory results during anti-TB treatment. Race/Ethnicity was self-reported according to the five Brazilian census categories (white, black, yellow, brown, or indigenous) [13] and, for the purposes of this study, was classified as white, African descent, east Asian descent, Mixed race (those with mixed racial ancestry, known as pardos), and Indigenous descent, as described elsewhere [14].

The cohort was followed for 3 years after the initiation of anti-TB treatment, using three sources of data: (*a*) medical records of patients participating in the Municipal TB Control Program; (*b*) Brazilian Ministry of Health information systems–the Notifiable Diseases Information System (SINAN) for TB and AIDS notification, the Mortality Information System (SIM), the Central Public Health Laboratory of Rio Grande do Sul, and the MDR-TB Information System; and (*c*) medical records of patients at the health care facilities where they were treated or monitored.

The following steps were performed for data search: (*i*) identification of the records of all 299 patients in the national database generated from the SINAN TB and AIDS databases; (*ii*) identification of the medical records of all patients in the Municipal TB Control Program and at the health care facilities where they were treated or monitored; and (*iii*) identification of the records of all patients in databases generated from the Mortality Information System, the Central Public Health Laboratory, and the MDR-TB Information System.

Data on smoking, alcohol consumption, and illicit drug use were extracted from patients’ medical records, and were not necessarily obtained from validated instruments. Clinical and laboratory monitoring data and anti-TB treatment results were extracted primarily from the SINAN databases. In some cases, however, these data were available only in the MDR-TB Information System or in patients’ records at reference facilities, such as the Outpatient Clinic at Hospital Sanatório Partenon, which is the center of excellence for treatment of DR-TB and MDR-TB in Porto Alegre.

According to Brazilian Ministry of Health guidelines on TB control, sputum smear microscopy should be performed monthly to monitor anti-TB treatment [11]. However, some cases were missing data on monthly smear results in the records or other information sources used. In these cases, because it was our intention to identify the month in which the patient’s sputum converted to negative, the sputum smear result from the following month was imputed to fill in for the missing value. In addition, because patients are unlikely to become positive after converting to negative, whenever a positive result was both preceded and followed by a negative result, this result was replaced with a negative result, as it was considered a possible human error in filling out the information. Thus, we sought to ensure a non-biased estimate of the main result of this evaluation regardless of the month of the negative result.

We checked the SINAN databases for data entered up to December 2010 in order to verify the occurrence of new (previously untreated) and relapse (previously treated) TB cases. TB relapse was defined as a patient who had become (and remained) negative while receiving treatment, but became smear-positive again after completion of treatment [11].

A database was created for the purposes of this study using EpiData 3.1 (EpiData Association, Odense, Denmark). Statistical analysis included the calculation of prevalence estimates, 95% confidence intervals, and group comparisons (resistant vs. non-resistant). Parametric and nonparametric analysis of longitudinal data (clinical and laboratory monitoring data of treated TB cases) was performed using Stata 10 (StataCorp LP, College Station, TX, USA). Values were considered statistically significant if *p* < 0.05.

## Results

A total of 299 cases of smear-positive pulmonary TB were included in the study. Among these cases, the prevalence of DR-TB, monoresistant TB, and MDR-TB was 14.4%, 8.4%, and 4.7% respectively. Additionally, 32.0% of previously treated patients had DR-TB (24/75), 18.7% had monoresistant TB (14/75), and 12.0% had MDR-TB (9/75) (Table 1).

**Table 1:** Level of drug resistance of patients with pulmonary TB identified through the II National Anti-TB Drug Resistance Survey (2006–2007), Porto Alegre, southern Brazil (*n* = 299).

Previous treatment	No resistance			Resistance									
				DR-TB			Monoresistant TB			MDR-TB			Total
							*n*	%	95%CI				*n*
Yes	51	68.0	(57.2–78.8)	24	32.0	(21.2–42.8)	14	18.7	(9.6–27.7)	9	12.0	(4.5–19.5)	75
No	205	91.5	(87.9–95.2)	19	8.5	(4.8–12.1)	11	4.9	(2.0–7.8)	5	2.2	(0.3–4.2)	224
Total	256	85.6	(87.9–95.2)	43	14.4	(10.4–18.4)	25	8.4	(5.2–11.5)	14	4.7	(2.3–7.1)	299

DR-TB, drug-resistant tuberculosis; MDR-TB, multidrug-resistant tuberculosis; TB, tuberculosis.

Sociodemographic characteristics for the overall sample and stratified by level of drug resistance are described in Table 2. Most patients were white (62.3%) men (72.6%) aged 25 to 45 years (49.5%), with less than 8 years of education (72.1%). There was no association between DR-TB, monoresistant TB or MDR-TB status and sociodemographic variables (*p* = 0.05).

**Table 2:** Sociodemographic characteristics of the study sample, stratified by level of anti-TB drug resistance, Porto Alegre, southern Brazil (2006–2007) (*n* = 299).

Characteristics	No resistance			Resistance											
				DR-TB			Monoresistant TB			MDR-TB			Total		
							*n*	%^a^	95%CI						
Sex															
Male	188	73.4	(64.8–82.1)	29	67.4	(53.4–81.4)	11	64.7	(42–87.4)	10	71.4	(47.8–95.1)	217	72.6	(67.5–77.6)
Female	68	26.6	(17.9–35.2)	14	32.6	(18.6–46.6)	6	35.3	(12.6–58)	4	28.6	(4.9–52.2)	82	27.4	(22.4–32.5)
Age, years															
18 to 25	53	20.8	(12.8–28.7)	6	14.0	(7.2–20.7)	2	11.8	(5.4–18.1)	3	21.4	(13.4–29.5)	59	19.8	(12–27.6)
26 to 35	73	28.6	(23.1–37.5)	10	23.3	(10.6–31.5)	3	17.6	(0–25.1)	3	21.4	(0–29.5)	83	27.9	(22.8–36.6)
36 to 45	53	20.8	(15.8–25.8)	12	27.9	(14.5–41.3)	5	29.4	(7.8–51.1)	5	35.7	(10.6–60.8)	65	21.8	(17.1–26.5)
46 to 55	51	20.0	(15.1–24.9)	9	20.9	(8.8–33.1)	4	23.5	(3.4–43.7)	2	14.3	(0–32.6)	60	20.1	(15.6–24.7)
56 to 65	14	5.5	(2.7–8.3)	6	14.0	(3.6–24.3)	3	17.6	(0–35.8)	1	7.1	(0–20.6)	20	6.7	(3.9–9.6)
66 to 75	7	2.7	(0.7–4.8)	0	0.0	(0–0)	0	0.0	(0–0)	0	0.0	(0–0)	7	2.3	(0.6–4.1)
76 to 90	4	1.6	(0–3.1)	0	0.0	(0–0)	0	0.0	(0–0)	0	0.0	(0–0)	4	1.3	(0–2.6)
Not reported	1	–	–	0	–	–	0	–	–	0	–	–	1	–	–
Race/Ethnicity															
White	148	61.9	(55.8–68.1)	27	64.3	(49.8–78.8)	14	82.4	(64.2–100)	6	46.2	(19.1–73.3)	175	62.3	(56.6–67.9)
African descent	64	26.8	(21.2–32.4)	13	31.0	(17–44.9)	2	11.8	(0–27.1)	7	53.8	(26.7–80.9)	77	27.4	(22.2–32.6)
Mixed	26	10.9	(6.9–14.8)	2	4.8	(0–11.2)	1	5.9	(0–17.1)	0	0.0	(0–0)	28	10.0	(6.5–13.5)
Asian descent	1	0.4	(0–1.2)	0	0.0	(0–0)	0	0.0	(0–0)	0	0.0	(0–0)	1	0.4	(0–1.1)
Not reported	17	–	–	1	–	–	0	–	–	1	–	–	18	–	–
Level of education															
None	14	5.9	(2.9–8.9)	2	4.8	(0–11.2)	2	11.8	(0–27.1)	0	0.0	(0–0)	16	5.7	(3–8.4)
1 to 3 years	49	20.6	(15.5–25.7)	7	16.7	(5.4–27.9)	4	23.5	(3.4–43.7)	3	23.1	(0.2–46)	56	20.0	(15.3–24.7)
4 to 7 years	108	45.4	(39.1–51.7)	22	52.4	(37.3–67.5)	9	52.9	(29.2–76.7)	5	38.5	(12–64.9)	130	46.4	(40.6–52.3)
8 to 11 years	57	23.9	(18.5–29.4)	11	26.2	(12.9–39.5)	2	11.8	(0–27.1)	5	38.5	(12–64.9)	68	24.3	(19.3–29.3)
12 years or more	10	4.2	(1.7–6.8)	0	0.0	(0–0)	0	0.0	(0–0)	0	0.0	(0–0)	10	3.6	(1.4–5.7)
Not reported	18	–	–	1	–	–	0	–	–	1	–	–	19	–	–
Institutionalization															
No	207	87.3	(83.1–91.6)	35	83.3	(72.1–94.6)	13	76.5	(56.3–96.6)	11	84.6	(65–100)	242	86.7	(82.8–90.7)
Prison	10	4.2	(1.7–6.8)	2	4.8	(0–11.2)	1	5.9	(0–17.1)	0	0.0	(0–0)	12	4.3	(1.9–6.7)
Nursing home	2	0.8	(0–2)	1	2.4	(0–7)	0	0.0	(0–0)	1	7.7	(0–22.2)	3	1.1	(0–2.3)
Hospital	10	4.2	(1.7–6.8)	2	4.8	(0–11.2)	1	5.9	(0–17.1)	1	7.7	(0–22.2)	12	4.3	(1.9–6.7)
Homeless	4	1.7	(0–3.3)	2	4.8	(0–11.2)	2	11.8	(0–27.1)	0	0.0	(0–0)	6	2.2	(0.4–3.9)
Other	4	1.7	(0–3.3)	0	0.0	(0–0)	0	0.0	(0–0)	0	0.0	(0–0)	4	1.4	(0–2.8)
Not reported	19	–	–	1	–	–	0	–	–	1	–	–	20	–	–
Employment															
Not employed	17	11.9	(6.6–17.2)	4	13.8	(1.2–26.3)	2	18.2	(0–41)	1	12.5	(0–35.4)	21	12.2	(7.3–17.1)
Informal	44	30.8	(23.2–38.3)	9	31.0	(14.2–47.9)	4	36.4	(7.9–64.8)	1	12.5	(0–35.4)	53	30.8	(23.9–37.7)
Formal	82	57.3	(49.2–65.4)	16	55.2	(37.1–73.3)	5	45.5	(16–74.9)	6	75.0	(45–100)	98	57.0	(49.6–64.4)
Not reported	113	–	–	14	–	–	6	–	–	6	–	–	127	–	–
Alcoholism															
Yes	77	40.5	(33.5–47.5)	14	40.0	(23.8–56.2)	5	41.7	(13.8–69.6)	4	36.4	(7.9–64.8)	91	40.4	(34–46.9)
No	113	59.5	(52.5–66.5)	21	60.0	(43.8–76.2)	7	58.3	(30.4–86.2)	7	63.6	(35.2–92.1)	134	59.6	(53.1–66)
Not reported	66	–	–	8	–	–	5	–	–	3	–	–	74	–	–
Smoking															
Yes	108	55.7	(48.7–62.7)	17	51.5	(34.5–68.6)	6	50.0	(21.7–78.3)	6	60.0	(29.6–90.4)	125	55.1	(48.6–61.5)
No	86	44.3	(37.3–51.3)	16	48.5	(31.4–65.5)	6	50.0	(21.7–78.3)	4	40.0	(9.6–70.4)	102	44.9	(38.5–51.4)
Not reported	62	–	–	10	–	–	5	–	–	4	–	–	72	–	–
Illicit drug use															
Yes	46	25.0	(18.7–31.3)	10	34.5	(17.2–51.8)	3	33.3	(2.5–64.1)	5	50.0	(19–81)	56	26.3	(20.4–32.2)
No	138	75.0	(68.7–81.3)	19	65.5	(48.2–82.8)	6	66.7	(35.9–97.5)	5	50.0	(19–81)	157	73.7	(67.8–79.6)
Not reported	72	–	–	14	–	–	8	–	–	4	–	–	86	–	–

DR-TB, drug-resistant tuberculosis; MDR-TB, multidrug-resistant tuberculosis; TB, tuberculosis.

^a^ Percentages were calculated using the number of reported cases (i.e., the total number of cases minus the number of unreported cases) as the denominator.

Table 3 shows patient clinical data for the sample as a whole and stratified by level of drug resistance. The most common signs and symptoms were productive cough (80.4%), weight loss (69.8%), and fever (41.3%). The most common comorbidity was HIV infection (26.2%), followed by diabetes mellitus (5.2%) (Table 4). There was no association between clinical features and presence of comorbidities (*p* > 0.05).

**Table 3:** Clinical features of the study sample, stratified by level of anti-TB drug resistance, Porto Alegre, southern Brazil (2006–2007) (*n*  =  299).

Characteristics	No resistance			Resistance											
				DR-TB			Monoresistant TB			MDR-TB			Total		
							*n*	%^a^	95%CI						
Dry cough															
No	189	94.0	(90.8–97.3)	32	94.1	(86.2–100)	14	93.3	(80.7–100)	9	90.0	(71.4–100)	221	94.0	(91–97.1)
Yes	12	6.0	(2.7–9.2)	2	5.9	(0–13.8)	1	6.7	(0–19.3)	1	10.0	(0–28.6)	14	6.0	(2.9–9)
Not reported	55	–	–	9	–	–	2	–	–	4	–	–	64	–	–
Productive cough															
No	36	17.9	(12.6–23.2)	10	29.4	(14.1–44.7)	4	26.7	(4.3–49)	4	40.0	(9.6–70.4)	46	19.6	(14.5–24.6)
Yes	165	82.1	(76.8–87.4)	24	70.6	(55.3–85.9)	11	73.3	(51–95.7)	6	60.0	(29.6–90.4)	189	80.4	(75.4–85.5)
Not reported	55	–	–	9	–	–	2	–	–	4	–	–	64	–	–
Fatigue															
No	36	17.9	(12.6–23.2)	10	29.4	(14.1–44.7)	4	26.7	(4.3–49)	4	40.0	(9.6–70.4)	46	19.6	(14.5–24.6)
Yes	165	82.1	(76.8–87.4)	24	70.6	(55.3–85.9)	11	73.3	(51–95.7)	6	60.0	(29.6–90.4)	189	80.4	(75.4–85.5)
Not reported	55	–	–	9	–	–	2	–	–	4	–	–	64	–	–
Fever															
No	117	58.2	(51.4–65)	21	61.8	(45.4–78.1)	8	53.3	(28.1–78.6)	6	60.0	(29.6–90.4)	138	58.7	(52.4–65)
Yes	84	41.8	(35–48.6)	13	38.2	(21.9–54.6)	7	46.7	(21.4–71.9)	4	40.0	(9.6–70.4)	97	41.3	(35–47.6)
Not reported	55	–	–	9	–	–	2	–	–	4	–	–	64	–	–
Hemoptysis															
No	182	90.5	(86.5–94.6)	28	82.4	(69.5–95.2)	13	86.7	(69.5–100)	9	90.0	(71.4–100)	210	89.4	(85.4–93.3)
Yes	19	9.5	(5.4–13.5)	6	17.6	(4.8–30.5)	2	13.3	(0–30.5)	1	10.0	(0–28.6)	25	10.6	(6.7–14.6)
Not reported	55	–	–	9	–	–	2	–	–	4	–	–	64	–	–
Chest pain															
No	166	82.6	(77.3–87.8)	31	91.2	(81.6–100)	14	93.3	(80.7–100)	8	80.0	(55.2–100)	197	83.8	(79.1–88.5)
Yes	35	17.4	(12.2–22.7)	3	8.8	(0–18.4)	1	6.7	(0–19.3)	2	20.0	(0–44.8)	38	16.2	(11.5–20.9)
Not reported	55	–	–	9	–	–	2	–	–	4	–	–	64	–	–
Sweating															
No	166	82.6	(77.3–87.8)	31	91.2	(81.6–100)	14	93.3	(80.7–100)	8	80.0	(55.2–100)	197	83.8	(79.1–88.5)
Yes	35	17.4	(12.2–22.7)	3	8.8	(0–18.4)	1	6.7	(0–19.3)	2	20.0	(0–44.8)	38	16.2	(11.5–20.9)
Not reported	55	–	–	9	–	–	2	–	–	4	–	–	64	–	–
Weight loss															
No	56	27.9	(21.7–34.1)	15	44.1	(27.4–60.8)	9	60.0	(35.2–84.8)	4	40.0	(9.6–70.4)	71	30.2	(24.3–36.1)
Yes	145	72.1	(65.9–78.3)	19	55.9	(39.2–72.6)	6	40.0	(15.2–64.8)	6	60.0	(29.6–90.4)	164	69.8	(63.9–75.7)
Not reported	55	–	–	9	–	–	2	–	–	4	–	–	64	–	–

DR-TB, drug-resistant tuberculosis; MDR-TB, multidrug-resistant tuberculosis; TB, tuberculosis.

^a^ Percentages were calculated using the number of reported cases (i.e., the total number of cases minus the number of unreported cases) as the denominator.

**Table 4:** Comorbidities of the study sample stratified by level of anti-TB drug resistance, Porto Alegre, southern Brazil (2006–2007) (*n* = 299).

Comorbidities	No resistance			Resistance									Total		
				DR-TB			Monoresistant TB			MDR-TB					
							*n*	%^a^	95%CI						
Diabetes															
No	220	95.2	(92.5–98)	36	92.3	(83.9–100)	15	93.8	(81.9–100)	11	100.0	(100–100)	256	94.8	(92.2–97.5)
Yes	11	4.8	(2–7.5)	3	7.7	(0–16.1)	1	6.3	(0–18.1)	0	0.0	(0–0)	14	5.2	(2.5–7.8)
Not reported	25	–	–	4	–	–	1	–	–	3	–	–	29	–	–
Hypertension															
No	224	97.0	(94.8–99.2)	39	97.5	(92.7–100)	16	94.1	(82.9–100)	11	100.0	(100–100)	263	97.0	(95–99.1)
Yes	7	3.0	(0.8–5.2)	1	2.5	(0–7.3)	1	5.9	(0–17.1)	0	0.0	(0–0)	8	3.0	(0.9–5)
Not reported	25	–	–	3	–	–	0	–	–	3	–	–	28	–	–
HIV															
No	156	72.9	(66.9–78.9)	30	78.9	(66–91.9)	13	81.3	(62.1–100)	7	70.0	(41.6–98.4)	186	73.8	(68.4–79.2)
Yes	58	27.1	(21.1–33.1)	8	21.1	(8.1–34)	3	18.8	(0–37.9)	3	30.0	(1.6–58.4)	66	26.2	(20.8–31.6)
Not reported	42	–	–	5	–	–	1	–	–	4	–	–	47	–	–
Mental illness															
No	228	98.3	(96.6–100)	40	100.0	(100–100)	17	100.0	(100–100)	11	100.0	(100–100)	268	98.5	(97.1–100)
Yes	4	1.7	(0–3.4)	0	0.0	(0–0)	0	0.0	(0–0)	0	0.0	(0–0)	4	1.5	(0–2.9)
Not reported	24	–	–	3	–	–	0	–	–	3	–	–	27	–	–
CKD															
No	231	100.0	(100–100)	39	97.5	(92.7–100)	17	100.0	(100–100)	11	100.0	(100–100)	270	99.6	(98.9–100)
Yes	0	0.0	(0–0)	1	2.5	(0–7.3)	0	0.0	(0–0)	0	0.0	(0–0)	1	0.4	(0–1.1)
Not reported	25	–	–	3	–	–	0	–	–	3	–	–	28	–	–

CKD, chronic kidney disease; DR-TB, drug-resistant tuberculosis; MDR-TB, multidrug-resistant tuberculosis; TB, tuberculosis.

^a^ Percentages were calculated using the number of reported cases (i.e., the total number of cases minus the number of unreported cases) as the denominator.

**Fig 1:**
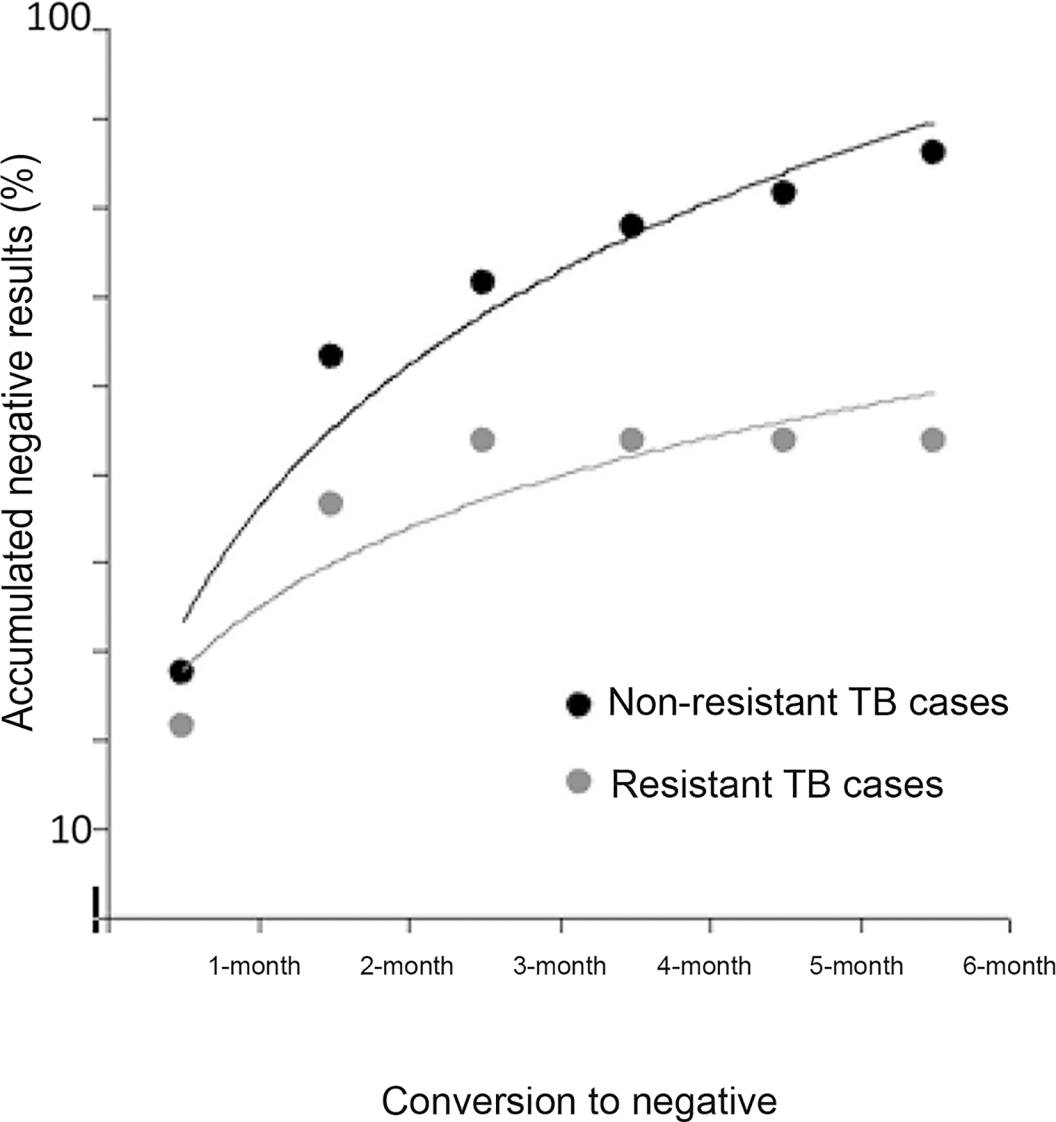
Monthly sputum conversion to negative in resistant vs. non-resistant tuberculosis (TB), Porto Alegre, Brazil (2006–2007).

Table 5 shows the results of clinical monitoring and anti-TB treatment outcomes for the sample as a whole and stratified by level of drug resistance. DR-TB and MDR-TB cases were significantly associated with occurrence of previously treated TB (*p* = 0.01). Overall, patients with DR-TB had worse clinical outcomes, as evaluated by laboratory tests, than patients with other levels of drug resistance (Table 5). Regarding sputum smear results, conversion to negative occurred in 81% of cases, and 65% of these patients achieved conversion at 2 months of treatment (Fig 1). However, only 45% of DR-TB cases became negative at 2 months (*p* = 0.004).

**Table 5:** Results of clinical monitoring and anti-TB treatment outcomes of the study sample, stratified by level of anti-TB drug resistance, Porto Alegre, southern Brazil (2006–2007) (*n* = 299).

Characteristics	No resistance			Resistance									Total		
				DR-TB			Monoresistant TB			MDR-TB					
							*n*	%^a^	95%CI						
No. of previous treatments															
One	34	14.4	(9.9–18.9)	16	40.0	(24.1–55.9)	8	50.0	(22.5–77.5)	5	38.5	(7.8–69.0)	50	18.1	(13.5–22.7)
Two	3	1.3	(0.1–2.7)	3	7.5	(1.0–16.0)	0	0.0	(0.0–0.0)	3	23.1	(34.2–49.6)	6	2.2	(0.4–3.9)
Three or more	9	3.8	(1.3–6.2)	2	5.0	(2.0–12.0)	1	6.3	(0.0–19.5)	1	7.7	(0.0–24.4)	11	4.0	(1.7–6.3)
No treatment	190	80.5	(75.4–85.5)	19	47.5	(31.3–63.7)	7	43.8	(16.4–71.0)	4	30.8	(1.7–59.7)	209	75.7	(70.6–80.8)
Not reported	20	–	–	3	–	–	1	–	–	1	–	–	23	–	–
Time of sputum conversion to negative															
Month 1	47	27.5	(20.7–34.2)	6	21.4	(5.2–37.6)	2	22.2	(0.0–56.1)	1	10.0	(0.0–32.6)	53	26.6	(20.4–32.8)
Month 2	61	35.7	(28.4–42.9)	7	25.0	(7.9–42.1)	2	22.2	(0.0–56.1)	4	40.0	(3.0–76.9)	68	34.2	(27.5–40.8)
Month 3	14	8.2	(4.0–12.3)	2	7.1	(0.0–17.3)	1	11.1	(0.0–36.7)	1	10.0	(0.0–32.6)	16	8.0	(4.2–11.8)
Month 4	11	6.4	(2.7–10.1)	0	0.0	(0.0–0.0)	0	0.0	(0.0–0.0)	0	0.0	(0.0–0.0)	11	5.5	(2.3–8.7)
Month 5	6	3.5	(0.7–6.2)	0	0.0	(0.0–0.0)	0	0.0	(0.0–0.0)	0	0.0	(0.0–0.0)	6	3.0	(0.6–5.4)
Month 6	8	4.7	(1.4–7.8)	0	0.0	(0.0–0.0)	0	0.0	(0.0–0.0)	0	0.0	(0.0–0.0)	8	4.0	(1.2–6.7)
No conversion	24	14.0	(8.8–19.3)	13	46.4	(26.7–66.1)	4	44.4	(3.9–84.9)	4	40.0	(3.0–76.9)	37	18.6	(13.1–24.0)
Not reported	85	–	–	15	–	–	8	–	–	4	–	–	100	–	–
Treat. outcomes															
Cure	166	69.8	(64.8–76.5)	19	45.2	(29.5–60.9)	6	35.2	(10.0–60.6)	5	38.5	(7.9–69.1)	185	66.7	(61.2–72.4)
Default	50	21.3	(16.0–26.5)	9	21.4	(8.5–34.4)	5	29.4	(5.3–53.5)	3	23.1	(3.4–49.5)	59	21.2	(16.4–26.1)
Transfer	3	1.3	(0.0–2.7)	2	4.8	(1.9–11.5)	1	5.9	(0.0–18.3)	1	7.7	(0.0–24.4)	5	1.8	(0.2–3.3)
Failure	1	0.4	(0.0–1.3)	7	16.7	(4.9–28.4)	1	5.9	(0.0–18.3)	3	23.1	(0.0–49.6)	8	2.8	(0.9–4.9)
Ongoing	1	0.4	(0.0–1.3)	0	0.0	(0.0–0.0)	0	0.0	(0.0–0.0)	0	0.0	(0.0–0.0)	1	0.4	(0.0–1.0)
Death	14	5.9	(2.9–9.0)	5	11.9	(1.7–22.1)	4	23.5	(1.0–46.0)	1	7.7	(0.0–24.4)	19	6.8	(3.9–9.8)
Not reported	3	–	–	0	–	–	0	–	–	0	–	–	3	–	–
Not notified^b^	18	–	–	1	–	–	0	–	–	1	–	–	19	–	–
No. of treatments after the survey period (relapse)															
One	32	13.5	(9.1–17.9)	3	7.1	(0.0–15.3)	1	5.9	(0.0–18.3)	1	7.70	(0.0–24.4)	35	12.5	(8.6–16.4)
Two	5	2.1	(0.3–3.9)	3	7.1	(0.0–15.3)	0	0.0	(0.0–0.0)	3	23.08	(0.0–49.6)	8	2.9	(0.9–4.8)
Three or more	4	1.7	(0.0–3.3)	0	0.0	(0.0–0.0)	0	0.0	(0.0–0.0)	0	0.00	(0.0–0.0)	4	1.4	(0.0–2.8)
None	196	82.7	(77.8–87.5)	36	85.7	(74.7–96.7)	16	94.1	(81.6–100.0)	9	69.23	(40.2–98.3)	232	83.1	(78.7–87.6)
Not reported	19	–	–	1	–	–	0	–	–	1	–	–	20	–	–
Total	256			43			17			14			299		

DR-TB, drug-resistant tuberculosis; MDR-TB, multidrug-resistant tuberculosis; TB, tuberculosis.

^a^ Percentages were calculated using the number of reported cases (i.e., the total number of cases minus the number of unreported cases) as the denominator.

^b^ Patients not found in the Brazilian Ministry of Health Notifiable Diseases Information System (SINAN) for TB and AIDS.

Regarding anti-TB treatment outcomes, cure was achieved in 66.7% of patients, and the default rate was 21.2% (Table 5). None of the patients with DS-TB or DR/MDR-TB was treated with directly observed therapy (DOT). There was a negative association between cure and presence of DR and monoresistant TB (*p* < 0.001). Overall, 16.9% of cases had at least one relapse. There was a trend toward a higher number of relapses among MDR-TB cases (30.7%) as compared with patients with other levels of drug resistance (17.3%) (Table 5).

## Discussion

This study evaluated the clinical features and treatment outcomes of 299 TB cases identified in Porto Alegre through the II National Anti-Tuberculosis Drug Resistance Survey conducted in Brazil (2006–2007). The results indicate that two-thirds of the patients achieved sputum smear conversion at 2 months, but DR-TB and MDR-TB cases showed lower conversion rates than drug-sensitive TB cases. After a 3-year follow-up, the cohort showed a high overall relapse rate (16.9%), which was even higher among patients with MDR-TB (30.7%).

Sputum monitoring and culture conversion have been shown to be good indicators of treatment outcome in drug-sensitive TB, but those indicators have not been validated for MDR-TB [15–17]. Among MDR-TB patients, persistent positive sputum cultures at month 6 of treatment had a high negative predictive value for failure and relapse, but only a modest positive predictive value (< 60%) [15,16]. Following WHO recommendations [3], we used sputum conversion at month 6 of treatment of MDR-TB, which may be useful where health services have limited and/or delayed access to culture results. Horne et al. analyzed 20 studies where drug sensitivity testing (DST) was available, and found that both sputum-smear microscopy and mycobacterial culture during TB treatment have low sensitivity and modest specificity for predicting failure and relapse [15]. Brust et al. [18], evaluating a cohort of 56 patients with MDR-TB from a rural area of South Africa, found that the only independent predictor of culture conversion at 6 months was smear positivity.

The sputum conversion rate observed in the entire sample was considered good in view of the low rate of adherence to this strategy in Porto Alegre [19], despite the Brazilian Ministry of Health recommendation that sputum smear examination be performed monthly (or at least at 2, 4, and 6 months) in addition to monthly clinical monitoring of anti-TB treatment [8].

The high relapse rates observed are well above the Brazilian Ministry of Health estimate of 10% [8]. In addition, these rates are much higher than those reported in other nationwide studies involving drug-susceptible TB patients (Porto Alegre, 4.5%; Rio de Janeiro, 8.0%) [20,21].

Also concerning was the high rate of default among patients with MDR-TB (23.1%). This rate is similar to that described by Tockzek et al. [22] when evaluating 10 studies in which directly observed therapy was not employed (26%).

Interruption of treatment may increase not only morbidity and mortality, but also the risk of transmission of resistant bacilli to healthy household members or even to institutionalized populations. As for cure, lower rates were found in patients with DR-TB and MDR-TB, which is consistent with the results reported in the literature [3,5,6,22].

The study sample consisted mainly of white male young adults with low levels of education. These sociodemographic characteristics are similar to those reported in previous studies [4,5,23,24].

Relatively high rates of smoking (55.1%), alcohol consumption (40.4%), and illicit drug use (26.3%) were observed in the current study. The Brazilian Ministry of Health estimates that over 20% of incident TB cases may be attributed to active smoking [11]. However, no evidence for an association of MDR-TB with smoking was found in the literature. The rate of alcohol consumption, although high, was similar to that reported in different regions of Brazil (Rio de Janeiro, 24.6%; Santa Catarina, 36.8%; Espírito Santo, 59.5%) [25–27] and in international series (South India, 29%) [28]. The literature suggests an association between TB and alcoholism, but evidence for an association between alcohol consumption and DR-TB is lacking [29]. Regarding illicit drug use, information on the relationship between injection drug use and DR-TB or MDR-TB is scarce [26,30,31]. In this study, these data were collected from patients’ medical records, which precluded assessment of the actual number of patients who used inhaled or injected drugs for a proper comparison. Therefore, no association was identified with those variables, as risk associated to smoking, alcoholism and illicit drug use varies depending on use characteristics, and this information was not available.

As for comorbidities, a high rate of HIV infection was observed among patients with MDR-TB (30.0%). Studies have demonstrated that HIV infection increases the likelihood of anti-TB treatment failure, but there is still no consensus on the relationship between HIV infection and increased transmission of multidrug-resistant strains of *M*. *tuberculosis* [4,32,33]. No association was found between presence of diabetes and level of drug resistance, despite reports indicating that this comorbidity is more common in DR-TB cases [6,34]. Although a higher frequency of chronic kidney disease was detected in DR-TB cases, the descriptive and exploratory nature of this research means we cannot provide confirmatory evidence for this association.

Despite the Brazilian Ministry of Health recommendation for directly observed therapy (DOT) as the standard of care for TB in Brazil [11], none of the patients with DS-TB or DR/MDR-TB was treated with DOT (data not shown). In addition, of 14 patients with MDR-TB, five (35.7%) came from hospitals (data not shown). Data from Porto Alegre Municipal Health Department epidemiological reports show that about 35% of patients who initiate anti-TB treatment come from hospitals [35], i.e., many patients use the emergency department as a point of entry into the TB control program. This is clearly not a desirable situation, because it is known that symptomatic patients who seek emergency care are often debilitated by prolonged illness, which increases the likelihood of transmitting drug-resistant strains in an environment without effective infection control measures.

Strengths of our study include: a) standardized screening of presumed DR-TB patients enrolled from eight public health care facilities (five primary health care units and three hospitals) in Porto Alegre; b) cultures and DST were performed at a reference laboratory that follows standard WHO guidelines; and c) the personnel performing cultures and DST were unaware of patients’ clinical or radiographic findings.

The main limitations of this study are the nature of data collection, as most of the data were originally collected through the nationwide survey or extracted from medical records, and the fact that these data were not collected under clinical research conditions, in which all variables are usually controlled. Additionally, we relied on a small sample size of drug-resistant TB cases in the study period.

In summary, this study attempted to add to the existing literature by further exploring the clinical features of patients with pulmonary TB according to the level of drug resistance at a specific setting (Porto Alegre, capital of the state of Rio Grande do Sul, southern Brazil), focusing on treatment outcomes in patients treated in routine clinical practice. Patients with DR-TB and MDR-TB had lower 2-month sputum conversion and cure rates and more relapses than non-resistant TB cases. These data may be used as a surveillance indicator, as they reflect regional differences and the low effectiveness of the TB control program in this particular geographical setting, highlighting the need for DOT, especially among patients under retreatment.
